# Implementing a template for major incident reporting: experiences from the first year

**DOI:** 10.1186/s13049-015-0132-0

**Published:** 2015-08-05

**Authors:** Sabina Fattah, Marius Rehn, Torben Wisborg

**Affiliations:** Department of Research and Development, Norwegian Air Ambulance Foundation, Drøbak, Norway; Anaesthesia and Critical Care Research Group, Faculty of Health Sciences, University of Tromsø, Tromsø, Norway; Field of Pre-hospital Critical Care, Network of Medical Sciences, University of Stavanger, Stavanger, Norway; London’s Air Ambulance, The Helipad, Royal London Hospital, Whitechapel, London UK; Department of Anaesthesiology and Intensive Care, Hammerfest Hospital, Finnmark Health Trust, Hammerfest, Norway; Norwegian National Advisory Unit on Trauma, Oslo University Hospital, Oslo, Norway

**Keywords:** Major incident, Reporting, Lessons learned

## Abstract

Major incidents are resource-demanding situations that require urgent and effective medical management. The possibility to extract learning from them is therefore important. Comparative analysis of information based on uniform data collection from previous incidents may facilitate learning. The Major Incident Reporting Collaborators have developed a template for reporting of the medical pre-hospital response to major incidents. The template is accompanied by an open access webpage (www.majorincidentreporting.org) for online reporting and access to published reports. This commentary presents the experiences from the first year of implementing the template including a presentation of the five published reports.

## Background

**Fig. 1:**
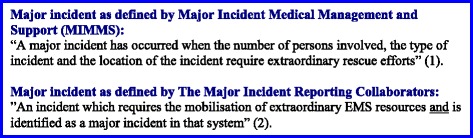
Definitions of major incidents

Major incidents are resource-demanding situations that require urgent and effective medical management. The available epidemiological data is heterogeneous and sparse, and thus of little help as a resource for planners. Major incidents comprise situations such as natural disasters, road traffic accidents, terrorist attacks and other causes of major trauma and medical morbidity. Given the unambiguity of major incident definitions [1, 2] (c.f Fig. 1) epidemiological data remains heterogeneous and no true incidence is described. A recent commentary asks “Why are we not learning from major incidents?” [3]. Comparative analysis of information based on uniform data collection from previous incidents may facilitate learning.

Several data templates with the aim of collecting uniform data from major incidents have been published [4]. In the absence of a feasibility-tested template especially focused on pre-hospital response, The Major Incident Reporting Collaborators developed a template for reporting of the medical pre-hospital response to major incidents [2]. The template is accompanied by an open access webpage (www.majorincidentreporting.org) for online reporting and access to published reports.

The aims of majorincidentreporting.org:

- To be a global open access online solution for both reporting and accessing reports describing the pre-hospital medical response to major incidents.
- Contribute to learning from previous incidents by comparative analysis of uniform data.
- Use such information to be better prepared and respond to the next incident in a more evidence-based manner.

What makes the template different from the pre-existing ones?

- Open access online reporting is available.
- All reports can be easily accessed at one location.
- Preliminary qualitative feasibility testing of the template has been undertaken.

### Confidentiality and data ownership

It was necessary to develop legal disclaimers for both the reporters and readers of reports, as the goal is global reporting with every country having its own legal situation. Before accessing the online reports readers must accept the terms and conditions stating that the authors are responsible for their content.

Patient confidentiality is especially sensitive in highly profiled cases with few patients involved and where patient information is available in the public domain through media and official reports. Due to the management of the webpage currently being situated in Norway, and lack of internationally applicable laws on this field, the Norwegian Data Protection Authority was consulted. Their decision was that data on less than six persons should not be presented. This limitation regarding patient data has been implemented into the online template. To further ensure confidentiality the editorial board revises information in each report that could compromise patient confidentiality prior to publication.

### Presentation

Limited financial resources have been a challenge as the webpage is solely sponsored by a charity (the Norwegian Air Ambulance Foundation). The reports are accompanied by a map of the affected area to make orientation easier for those unfamiliar with the region and data is presented in the format of questions accompanied by the corresponding answer. Limitations exist with regard to making the reports suitable for journal publication without extensive revisions. However, using the systematic information in the online template as a framework for case report structure is emphasized, and the subsequent publication of a case report after submitting data through the template and website is encouraged.

### Future implementation

Both wider implementation and more secure funding are necessary for the further dissemination of the template and expand the existing technological solutions and manpower for maintaining the project. Anchoring the project under national health authorities is a main goal, as is the involvement of authorities responsible for other emergency responders such as police and fire services. The template is limited to primarily the pre-hospital medical response in a sudden-onset emergency. Development of sub-templates for reporting a small amount of data especially relevant for Helicopter Emergency Medical Services (HEMS), chemical, biological, radiological and nuclear (CBRN), police and fire response among others may be a possibility for making the template useful in a wider context.

### Challenges in dissemination

**Fig. 2:**
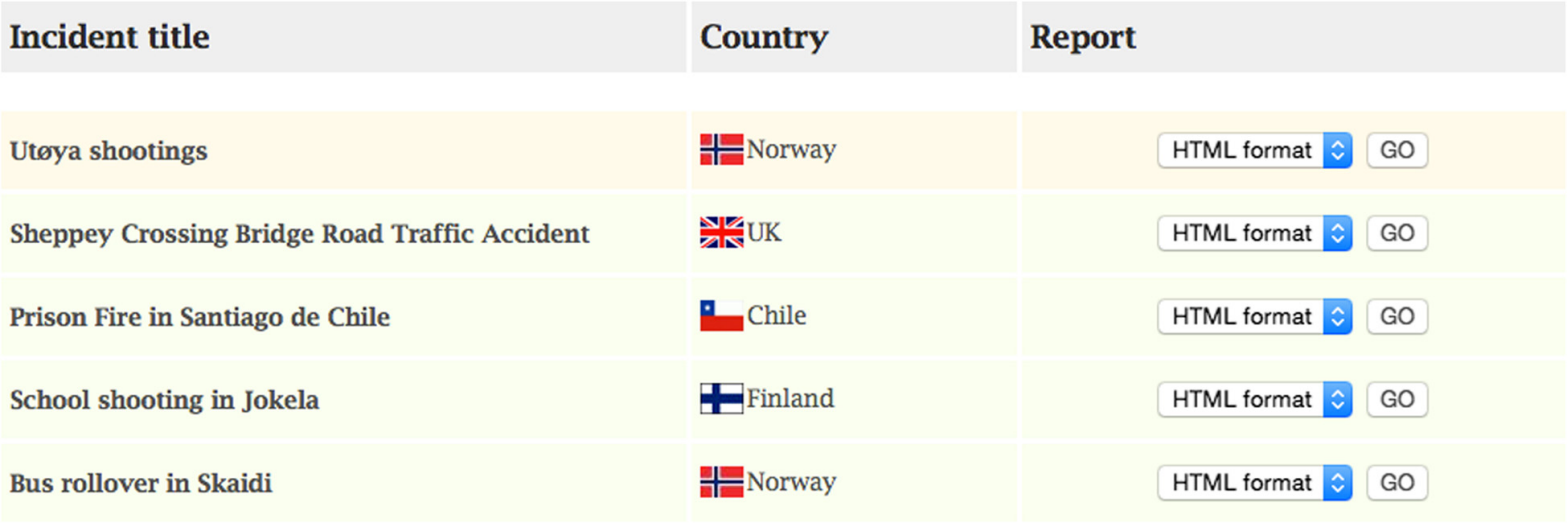
Overview of published reports on majorincidentreporting.org

The main challenge so far has been the time delay in receiving completed reports. Currently, five reports have been published (c.f Fig. 2, Table 1). Preliminary results from a qualitative feasibility study suggest a need for revision and reducing the volume of work necessary for completing a report (Fattah S, Agledahl KM, Rehn M, Wisborg T: Experience with a novel global open access template for major incidents: qualitative feasibility study. Submitted). Dissemination of published reports has been done through e-mails, twitter, facebook and commentaries [5], in addition to the website itself. As no formal obligation to report after major incidents exist, it should be underlined that it is considered a moral and ethical imperative to disseminate experiences and lessons learned after participating in major incidents, in the interest of future colleagues and victims. The template and website provides a mean for this.

**Table 1:** Overview of reports submitted to majorincidentreporting.org. All full-text reports are freely accessible in full-text at: http://www.majorincidentreporting.org/read.html#.VRuiQ4tx2pp

Reporter	Incident title	Summary	Example of a major lessons learned
Dr. Sollid *HEMS physician* Norway	Utøya shootings	• 21 July 2011	• On-going shooting caused delayed access to EMS. Therefore need for a better system for cooperating with police in incidents with on-going shooting.
		• Terrorist attack on youth at a political summer camp on the Island of Utøya.	
Dr. Hardy *A&E doctor* United Kingdom	Sheppey Crossing Bridge Road Traffic Accident	• 5 September 2013	• Successful triage performed by highly skilled paramedics avoided unnecessary patients being sent to hospital.
		• Road traffic accident involving 150 cars and 200 people occurred on the Sheppey Crossing Bridge in Kent.	
Dr. Cortes Picazo. *Director of EMS* Chile	Prison Fire in Santiago de Chile	• 8 December 2010	• Avoid overcrowding in prisons.
		• Fire in Prison of San Miguel with 1900 inmates and guards inside.	
		• Most of the victims died of inhalation of smoke or toxic gases and burns.	
Dr. Jama *Medical Director of EMS* Finland	School shooting in Jokela	• 7 November 2011	• Need for better communication between EMS and police. EMS was not allowed into the building as the shooter was still inside during the response phase.
		• Student opened fire on school premises and killed eight people.	
Dr. Iversen *HEMS physician* Norway	Buss roll over in Skaidi	• 19 November 2011	• As a strike of luck the first ambulance on-scene had the same triage system as the HEMS. Since the time this incident occurred a national standard for triage has been implemented in Norway.
		• Buss carrying 23 persons rolled over in Skaidi; a cottage area in mountainous inland of Finnmark County.	

### Future dissemination

For future dissemination to succeed the template will need to be translated into several languages allowing it to be accessible to the global community who are non-fluent in English. Translation into Spanish is underway. Currently, the project has three endorsing societies; London’s Air Ambulance, Norwegian National Advisory Unit on Trauma and the Norwegian Society for Trauma, Emergency and Disaster Medicine. A letter of support from the UK National Health Service (NHS) has been received and made available on www.majorincidentreporting.org. Endorsers do not offer financing, but help to spread the word of the template and its reporting possibilities. More endorsing societies are welcomed.

### Future research

Questions for potential future research could include; How can the data in the reports be useful for others? Do different persons report similarly from the same incident? What motivates people to report and how can we overcome hindrances in low rates of reporting? Is the template developed by European experts relevant for other settings? Comparison of response to and outcome of different incidents can also be a topic once more reports are received. The editorial board welcomes research initiatives seeking to use data from the database for non-profit research aimed at improving major incident preparedness and response.

## Conclusion

Implementation and dissemination of the major incident reporting template depends on submission of reports, and is thus a challenge. Reports have been submitted and further work is underway to translate it into Spanish and find long-term financing for the project. The template and webpage are non-profit, freely accessible tools to facilitate learning lessons from major incidents. Therefore, we hope colleagues will support this work by helping to disseminate information of it, submit reports and collaborate on research projects related to it.
